# Do elevations in temperature, CO_2_, and nutrient availability modify belowground carbon gain and root morphology in artificially defoliated silver birch seedlings?

**DOI:** 10.1002/ece3.665

**Published:** 2013-07-22

**Authors:** Liisa Huttunen, Karita Saravesi, Annamari Markkola, Pekka Niemelä

**Affiliations:** 1 Section of Ecology, Department of Biology, University of TurkuFI-20014, Turku, Finland; 2 Department of Biology, University of OuluP.O. Box 8000, FI-90014, Oulu, Finland; 3 Section of Biodiversity and Environmental Science, Department of Biology, University of TurkuFI-20014, Turku, Finland

**Keywords:** *Betula pendula*, climate change, fertilization, fine roots, folivory, plant sugar allocation

## Abstract

Climate warming increases the risk of insect defoliation in boreal forests. Losses in photosynthetically active surfaces cause reduction in net primary productivity and often compromise carbon reserves of trees. The concurrent effects of climate change and removal of foliage on root growth responses and carbohydrate dynamics are poorly understood, especially in tree seedlings. We investigated if exposures to different combinations of elevated temperature, CO_2_, and nutrient availability modify belowground carbon gain and root morphology in artificially defoliated 1-year-old silver birches (*Betula pendula*). We quantified nonstructural carbohydrates (insoluble starch as a storage compound; soluble sucrose, fructose, and glucose) singly and in combination in fine roots of plants under winter dormancy. Also the total mass, fine root proportion, water content, and length of roots were defined. We hypothesized that the measured properties are lower in defoliated birch seedlings that grow with ample resources than with scarce resources. On average, fertilization markedly decreased both the proportion and the carbohydrate concentrations of fine roots in all seedlings, whereas the effect of fertilization on root water content and dry mass was the opposite. However, defoliation mitigated the effect of fertilization on the root water content, as well as on the proportion of fine roots and their carbohydrate concentrations by reversing the outcomes. Elevation in temperature decreased and elevation in CO_2_ increased the absolute contents of total nonstructural carbohydrates, whereas fertilization alleviated both these effects. Also the root length and mass increased by CO_2_ elevation. This confirms that surplus carbon in birch tissues is used as a substrate for storage compounds and for cell wall synthesis. To conclude, our results indicate that some, but not all elements of climate change alter belowground carbon gain and root morphology in defoliated silver birch seedlings.

## Introduction

Global climate change has expanded the ranges and increased the population densities of insect pests in high latitude forests intensifying the risk of defoliation (Wolf et al. 2008; Jepsen et al. 2011). This is likely to reduce forest net primary productivity and, in the worst-case scenario, initiates tree deaths in wide areas (Kurz et al. 2008). However, trees' responses to foliar losses are mainly dependent on their physiological state, environmental conditions (e.g., temperature), and resource availability (water, CO_2_, and nutrients; Maschinski and Whitham 1989; Eyles et al. 2009). For instance, leaf regrowth following folivory requires viable meristems or buds that can be quickly activated, and carbohydrates as energy reserves to renew photosynthetically active surfaces (Henriksson et al. 1999; Collin et al. 2000; Sala et al. 2012). Here, the main energy source is stored starch that is broken down into mono- and disaccharides, and remobilized into expanding organs (El Zein et al. 2011). Although the renewed leaves become carbohydrate sources quite soon, after reaching 10–50% of their final size (Valjakka et al. 1999; Keel and Schädel 2010), the second flush of foliage often compromises the carbon reserves in such organs as bark and roots and thus disturbs the forthcoming growth (e.g., Kaitaniemi et al. 1999; Huttunen et al. 2012).

Irrespective of energy consumption (Niinemets 1999), second-flush leaves are an essential part of the trees' growth recovery and photosynthate storage. It is commonly known that these leaves can even have higher photosynthesis (e.g., Hoogesteger and Karlsson 1992; Turnbull et al. 2007) indicating improved ability to support carbon gain in root stores. On the other hand, accumulation of assimilates as carbon reserves is usually initiated in trees after growth termination late in the season (Landhäusser and Lieffers 2003; Wong et al. 2003; Gaugher et al. 2005; Regier et al. 2010). If fully grown trees or seedlings are defoliated in mid or late season, formation of new leaves reduces carbon allocation into roots by renewing shoot growth (Collin et al. 2000; Willaume and Pagès 2011; Landhäusser and Lieffers 2012), which has a tendency to inhibit many physiological processes, such as winter hardening (Wargo 1978; Thomas et al. 2004). It is also possible that trees suffering from severe defoliation late in the season do not produce any second-flush leaves; this may have a positive influence on their energy reserves available during spring growth resumption (Wargo 1991). Therefore, the timing and frequency of foliage damage are important factors affecting carbon gain and storage in different plant organs (Tuomi et al. 1989; Saravesi et al. 2008). However, although early spring starch reserves in roots tend to be reduced due to previous insect defoliation (Wargo 1991; Landhäusser and Lieffers 2012), winter maintenance respiration (Barbaroux et al. 2003; Zhu et al. 2012), or carbon allocation into different organs as cryoprotectants (Kasuga et al. 2007), the concentrations increase during summer; when new leaves have been generated, assimilates produced soon become suppliers of growing and storing organs (Landhäusser and Lieffers 2003; Gaugher et al. 2005; Keel and Schädel 2010). To support leaf and sprout formation, carbohydrates from distant lateral roots are also mobilized, although the transport is costly and affects root growth negatively (Vogt and Bloomfield 1991; Willaume and Pagès 2011).

The concurrent effects of climate change and removal of foliage on root morphology, carbohydrate dynamics, and growth are poorly understood, especially in tree seedlings. Usually elevations in both CO_2_ and temperature increase fine root productivity, respiration, and soil carbon efflux, as well as root turnover rates in different plant species (e.g., Edwards and Norby 1999; Pumpanen et al. 2012). All this is reflected in root tissues in terms of carbohydrate concentrations, water content, and growth responses; however, these responses to CO_2_ or temperature elevations are inconsistent, showing either significant increases or decreases in different plant species (Edwards and Norby 1999; Pregitzer et al. 2000; Olszyk et al. 2003; Drake et al. 2008). Moreover, the broad impacts of climate change include alterations in plant nutrient availability. Warming increases atmospheric deposition of, for example, nitrogen, and accelerates mineralization rates of organic nitrogen or phosphorus in soil by stimulating decay or rock weathering (Verburg 2005; Hole and Engardt 2008; Dijkstra et al. 2012). Likewise, increase in temperature heats and dries soils by accelerating evapotranspiration rates, which affect root water availability (Akinci and Lösel 2012; Houle et al. 2012). However, soil sensitivity to drought alters regionally; for example, in some areas of boreal forests, increased precipitation (Soja et al. 2007) and deeper and longer lasting frost due to thinner snow cover (Aphalo et al. 2006) may mitigate the effects of climate warming on the ground. In any case, plant responses at higher nutrients and lower water content are mixed: total nonstructural carbohydrate concentrations in roots decrease, as high nutrient availability shifts carbohydrate allocation toward aboveground organs reducing belowground productivity (Kobe et al. 2010; Kleczewski et al. 2012), whereas low water availability tends to affect roots conversely (Akinci and Lösel 2012). Plants that have experienced defoliation have also shown prioritized aboveground growth, similarly as with ample nutrients (Eyles et al. 2009).

In this study, we investigated if components characterized to climate change (i.e., different combinations of elevated temperature, CO_2_, and nutrient availability) modify the carbon gain, water content, and growth of belowground tissues in artificially defoliated, 1-year-old silver birch seedlings. Here, we reflect the plant responses to nitrogen availability; this element is one of the main factors limiting growth in boreal forest (Helmisaari et al. 2009), also for genus *Betula* (Ingestad and Lund 1979). We quantify nonstructural (soluble and storage) carbohydrates and their combinations in fine roots, and define the root morphological properties (dry mass, proportion of fine roots, water content, and length) of dormant plants in late winter. Previously, in the same system, we examined damage-induced aboveground responses of the same trees (see Huttunen et al. 2007, 2008). We hypothesized that the absolute and relative amounts of carbohydrates, the dry mass, the fine root proportion, the water content, and the length of roots are lower in those defoliated birch seedlings that grow with ample resources than in those with scarce resources. This is due to greater potential in assigning assimilates to aboveground compensation in high resource environments. In this case, intensified shoot compensatory growth following defoliation and the consequent delayed winter hardening lead to reduced carbon allocation and growth in belowground organs.

## Materials and Methods

### Plant material and treatments

**Figure 1:**
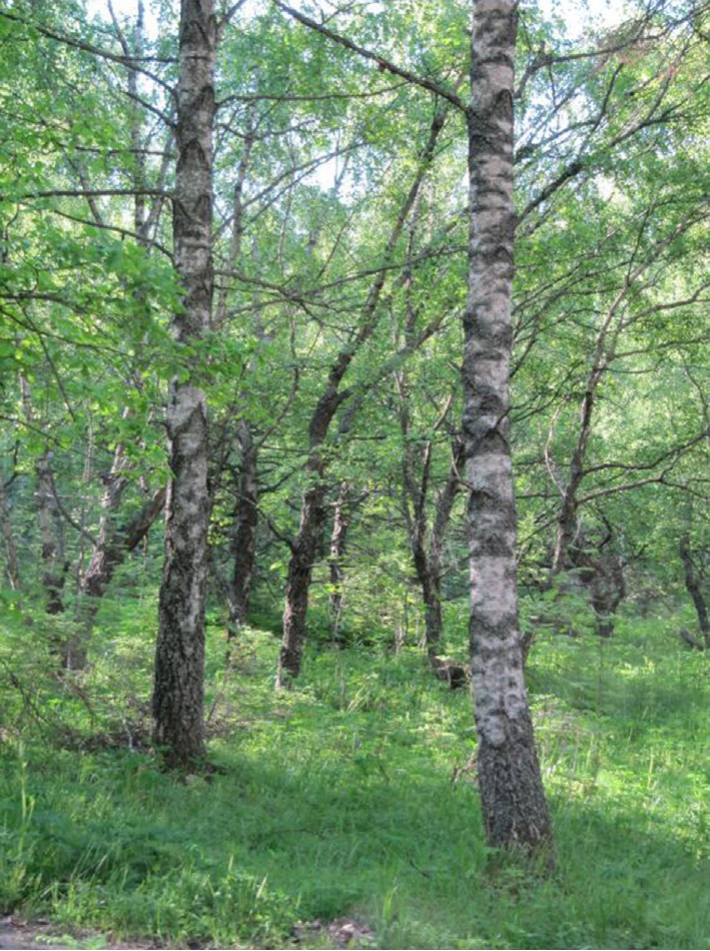
Mature cultivars of silver birches (*Betula pendula*) in southeastern Finland. Photo by Matti Rousi.

Throughout the summer 2002, silver birch seedlings were grown in trays with peat as the growth medium, and subjected to different abiotic conditions represented later on at the Mekrijärvi Research Station (62°47′N, 30°58′E), University of Eastern Finland. The plants were seed born with an origin of a naturally regenerated silver birch stand in southeastern Finland (61°48′N, 29°18′E). The juvenile stage of plants took place in an unheated greenhouse; after reaching a mean height of 6 cm, the seedlings were transferred to Mekrijärvi (Fig. 1).

The seedlings were exposed to climate treatments that were designed to correspond to the scenario of an increased temperature and a doubled atmospheric CO_2_ concentration in situ eastern Finland (Carter et al. 2002). These treatments prevailed in 16 outdoor chambers with the following settings: ambient temperature tracing the natural temperature outside with ambient CO_2_ (=360 parts per million, ppm = μmol/mol), ambient temperature with elevated CO_2_ (=720 μmol/mol), elevated temperature being +2°C higher than ambient with ambient CO_2_ and elevated temperature with elevated CO_2_. Each treatment was replicated in four chambers, and each chamber was equipped with computer-controlled sensing technology to monitor and maintain the temperature and CO_2_ at target levels (Kellomäki et al. 2000). However, temperature changes in plant growth medium were not monitored during the experiment, although it is commonly known that increasing atmospheric temperature may elevate soil temperature and improve root growth especially at 62°N, where the forest soil temperatures are typically low in summer time (Aphalo et al. 2006). To reduce the effect of within-chamber variation, the seedling trays were repositioned twice during the summer.

In each chamber, the seedlings were randomly assigned into two fertilizer treatments (48 seedlings per treatment) being 0 and 270 kg nitrogen (N) ha year^−1^ or 0 and 132.3 mg N per seedling, respectively. The latter was set to simulate the conditions prevailing in reforested arable lands under Finnish conditions after intensive practice of agriculture. Fertilizer was dissolved in water and applied weekly for 7 weeks starting in early June. The first three times the fertilizer was Kekkilä SuperX-9 (nitrogen, phosphorus, potassium; NPK: 19%–4%–20% of dry mass in respective order), and the following four times it was Kekkilä SuperX-5 (NPK: 12-5-27). Both the fertilizers contained balanced quantities of required mineral nutrients and trace elements for birch seedlings. Same volume of fertilizer, 126 mg per seedling with 23.9 or 15.1 mg N depending on the fertilizer was applied each time to maintain the prescribed levels of N. To prevent excessive dehydration, all the seedlings were irrigated daily throughout the summer. Detailed description of the different fertilizer treatments and seedling material is provided by Huttunen et al. (2007, 2008).

The seedlings under each fertilizer and climate treatment combination were randomly allocated to 0 or 50% defoliation, totaling 24 seedlings per treatment. Defoliation was conducted twice, on July 1st and 29th, by tearing the corresponding amount from the apical portion of each leaf (see Huttunen et al. 2007). The first tissue removal took place 4 weeks after commencement of fertilizer application; at the time, the seedlings had received 86.8 mg of N. The second tissue removal was conducted a week after termination of fertilizer application, and it was directed at those leaves that had developed along with height growth following the first tissue removal. According to elemental analysis performed on birch leaves in mid-August 2002, the nitrogen status of seedlings was affected by fertilizing: the N content was higher in fertilized (4.5 ± 0.05% dry weight [DW]; range 3.9–5.4% DW) than unfertilized seedlings (2.1 ± 0.08% DW; range 0.7–4.4% DW; see Huttunen et al. 2008).

In October 2002, all seedlings were dormant; their height growth was terminated, and bud formation and leaf abscission completed. This enabled their outdoor storage in an open shelter (10 m × 10 m, height 1.5 m), which was fenced with chicken wire and covered with a plastic tarpaulin to prevent damage by hares (*Lepus timidus*). Each seedling was positioned at random in the shelter. In January 2003, four seedlings per treatment combination per chamber (a total of 256 seedlings) were randomly selected and divided into roots and stems from the root collar. The stems were used in a separate experiment by a separate researcher. The root systems overwintered outdoors in pots until late March. At that time the roots were stored at +8°C until thawed. Defrosted peat was carefully cleaned away and thereafter the roots were allocated to different analyses. A total of 64 root systems (*n* = 4 in each defoliation, fertilization, and climate treatment combination) were frozen at −20°C for carbohydrate analyses, and 192 root systems (*n* = 12 in each treatment combination) were assigned for determinations of morphological properties, that is, root fresh and dry mass, water content, and main root length, which was measured down from the root collar. After fresh mass and root length measurements, the 192 root systems were freeze dried and divided into coarse (>1 mm in diameter) and fine roots (<1 mm in diameter). Thereafter, their dry mass and water content were determined.

### Root carbohydrate analyses

Fine roots (<1 mm in diameter) were harvested from the 64 root systems, freeze dried, and homogenized into powder with a ball mill. Thereafter, an enzymatic colorimetric assay was employed to quantify the absolute and relative masses of nonstructural carbohydrates in root material. These carbohydrates were soluble mono- and disaccharides (glucose, fructose, and sucrose) and an insoluble polysaccharide, starch. Here, the disaccharide sucrose is characterized as the main photoassimilate transporter in many plants (Wind et al. 2010), whereas starch is the major storage compound; it is typically found, for example, in roots of deciduous trees (Regier et al. 2010). Although birch tissues contain a variety of carbohydrates (e.g., xylitol, a sugar alcohol that derives from polysaccharide xylan), the analyzed sugars were chosen due to their nature of being profusely involved in growth resumption and primary metabolism (Gaugher et al. 2005; Lachke 2006). The mono- and disaccharides were extracted in water (2.5 h at +60°C), whereas starch was extracted in dimethyl sulphoxide (40 min at +60°C). Glucose, fructose, and sucrose were quantified directly, where starch was first hydrolyzed by amyloglucosidase and then quantified as glucose using a protocol described by Beutler et al. (1978). The relative concentrations of nonstructural carbohydrates were expressed as a percentage of dry weight of fine roots (% DW).

### Statistical analyses

The statistical analyses were carried out by SAS 9.3 software (SAS Institute Inc., Cary, NC). A linear mixed-effects model analyses of variance (ANOVA) for split-plot design was used to analyze the effects of temperature (T), CO_2_, fertilization (F), defoliation (D), and their interactions on the root dry mass (g DW), the fine root proportion as a fraction of total root mass (% DW), the absolute (g) and relative (%) root water contents, the main root length, and the absolute (g DW) and relative (% DW) amounts of carbohydrates in fine roots of silver birch seedlings. All the analyzed carbohydrates are presented as percentage of dry mass. The total nonstructural carbohydrates (TNCs; combination of starch, sucrose, fructose, and glucose) and starch are also presented as g DW, which was obtained by multiplying the fine root mass (g DW) by the carbohydrate concentration (% DW) and dividing the outcome by 100. The statistical model included the explanatory variables and their interactions as fixed effects, and individual seedling and chamber as random effects. Restricted maximum likelihood (REML) method was used to estimate the variance and covariance components. The pairwise comparisons were executed using the Tukey's test. The Kenward–Roger method was employed to estimate the degrees of freedom. Based on Akaike information criteria and Akaike weights, the chosen covariance structure was variance components, which was incorporated into the model. To prevent underestimates in error variances and thus less powerful tests, a no-bound option was used. This removed the lower bound of zero from the variance components. To analyze root carbohydrates within each treatment combination the number of replicates (i.e., roots) was four (*n* = 4). To analyze root morphological properties, it was 12 (*n* = 12). The critical alpha was <0.1. Throughout the results, the estimated marginal means (i.e., the least squares means in SAS) with the standard errors obtained from the statistical models are given for the measured parameters.

## Results

### Root morphological properties; total dry mass (g DW), proportion of fine roots (g DW and% DW), water content (g), and main root length (cm)

**Figure 2:**
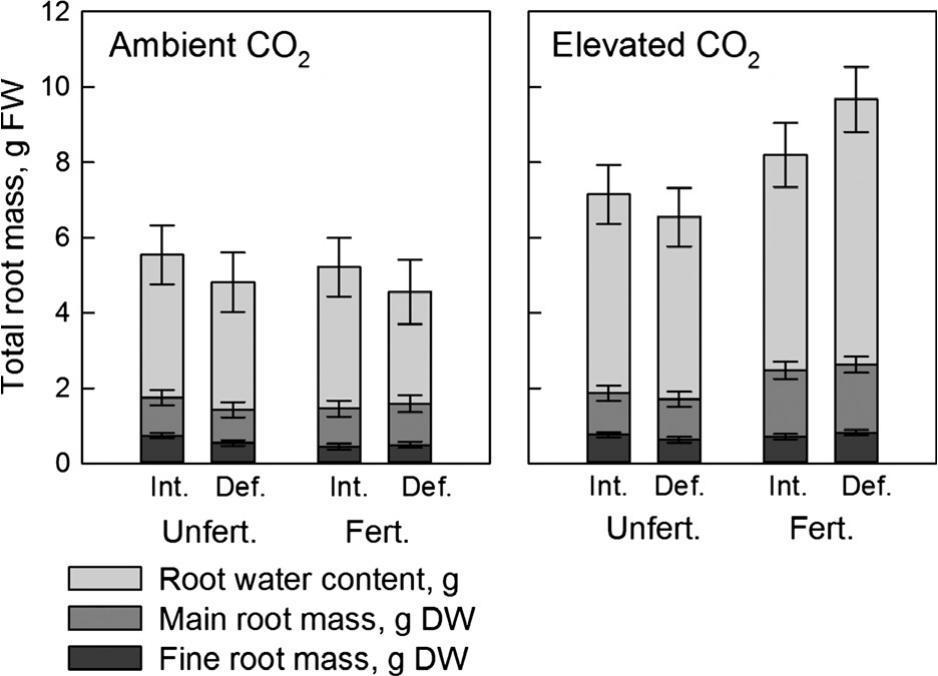
Total root mass (g fresh mass, FW) of silver birch seedlings in different treatment combinations. Estimated marginal means with ±SE are presented as histograms that are divided into water content (g) and shares of fine and main roots (g DW). Root samples for the analysis were collected in late March 2003. Int., intact seedling; Def., defoliated seedling; Unfert., unfertilized seedling; Fert., fertilized seedling.

In all seedlings, total dry mass of roots (dry mass of coarse, i.e., main roots + dry mass of fine roots) ranged between 0.24 and 6.91 g. The highest mass, 2.55 ± 0.18 g DW, was recorded in seedlings that were fertilized and subjected to elevated CO_2_ treatment (Table 1, Fig. 2). In contrast, the average root mass was lower in fertilized than unfertilized seedlings under ambient CO_2_, 1.52 ± 0.17 g DW versus 1.59 ± 0.16 g DW, respectively.

**Table 1:** Summary of linear mixed model analysis results for the effects of temperature, CO_2_, fertilization, and defoliation and their interactions on root morphological properties of silver birch seedlings

Source	Total root mass, g DW				Fine root mass, g DW				Proportion of fine roots, % DW			
		
	Num df	Den df	*F*	*P*	Num df	Den df	*F*	*P*	Num df	Den df	*F*	*P*
CO_2_	1	12.1	9.30	**0.010**	1	12	4.73	**0.051**	1	11.9	0.25	0.629
Temperature (T)	1	12.1	0.81	0.385	1	12	1.27	0.282	1	11.9	3.22	**0.098**
CO_2_ × T	1	12.1	0.16	0.698	1	12	0.06	0.806	1	11.9	0.01	0.942
Fertilization (F)	1	150.0	7.14	**0.008**	1	149	1.77	0.185	1	150.0	41.67	**<0.001**
Defoliation (D)	1	150.0	0.17	0.681	1	149	1.19	0.278	1	149.0	2.70	0.103
CO_2_ × F	1	150.0	10.23	**0.002**	1	149	9.75	**0.002**	1	150.0	0.01	0.913
CO_2_ × D	1	150.0	0.13	0.722	1	149	0.61	0.438	1	149.0	0.74	0.391
T × F	1	150.0	1.50	0.222	1	149	0.41	0.521	1	150.0	1.17	0.280
T × D	1	150.0	1.26	0.263	1	149	0.21	0.649	1	149.0	6.04	**0.015**
F × D	1	149.0	2.17	0.143	1	148	10.13	**0.002**	1	149.0	3.49	**0.064**
CO_2_ × T × F	1	150.0	0.16	0.691	1	149	0.81	0.370	1	150.0	0.57	0.450
CO_2_ × T × D	1	150.0	0.90	0.346	1	149	0.75	0.387	1	149.0	0.56	0.454
CO_2_ × F × D	1	149.0	0.05	0.816	1	148	<0.01	0.956	1	149.0	1.39	0.241
T × F × D	1	149.0	0.51	0.476	1	148	0.31	0.580	1	149.0	0.05	0.832
CO_2_ × T × F × D	1	149.0	0.18	0.674	1	148	0.38	0.537	1	149.0	0.16	0.694

	Root water content, g				Root water content, %				Root length, cm			
CO_2_	1	12.3	15.34	**0.002**	1	12.1	0.79	0.392	1	12.6	8.31	**0.013**
Temperature (T)	1	12.3	2.62	0.131	1	12.1	1.01	0.335	1	12.6	0.13	0.721
CO_2_ × T	1	12.3	0.20	0.664	1	12.1	0.20	0.662	1	12.6	0.62	0.445
Fertilization (F)	1	150	11.20	**0.001**	1	148.0	3.88	**0.051**	1	159.0	16.79	**<0.001**
Defoliation (D)	1	149	1.18	0.279	1	148.0	1.62	0.205	1	159.0	0.14	0.706
CO_2_ × F	1	150	7.77	**0.006**	1	148.0	0.76	0.386	1	159.0	1.56	0.214
CO_2_ × D	1	149	0.01	0.941	1	148.0	0.93	0.337	1	159.0	0.07	0.799
T × F	1	150	0.12	0.728	1	148.0	2.23	0.138	1	159.0	0.92	0.340
T × D	1	149	0.02	0.880	1	148.0	0.55	0.459	1	159.0	0.13	0.718
F × D	1	149	1.08	0.300	1	148.0	3.74	**0.055**	1	159.0	0.01	0.936
CO_2_ × T × F	1	150	0.60	0.440	1	148.0	0.39	0.536	1	159.0	0.05	0.823
CO_2_ × T × D	1	149	0.16	0.688	1	148.0	0.19	0.660	1	159.0	0.26	0.610
CO_2_ × F × D	1	149	3.14	**0.078**	1	148.0	4.40	**0.038**	1	159.0	0.41	0.525
T × F × D	1	149	<0.01	0.974	1	148.0	0.82	0.366	1	159.0	0.05	0.828
CO_2_ × T × F × D	1	149	0.02	0.888	1	148.0	<0.01	0.952	1	159.0	2.34	0.128

Proportion of fine roots is presented as a fraction of total root dry mass (% DW). The significant *P* values (<0.1) are given in bold. Num df, numerator degrees of freedom; Den df, denominator degrees of freedom.

The proportion of fine roots ranged between 15.8 and 74.6% of total root dry mass, or between 0.13 and 1.57 g DW, and showed a decrease in those seedlings that were defoliated and subjected to elevated temperature (Table 1; proportion of fine roots, % DW, T × D effect), or defoliated without fertilization (Table 1, Fig. 2; the F × D effect in absolute fine root mass). Fertilizing alone lowered the proportion of fine roots in all seedlings (Table 1).

The root water content in all seedlings ranged between 0.6 and 15. 8 g, or 26.7 and 92.3% from fresh root mass. Typically, fertilization increased the water content (Table 1). Defoliation lowered the absolute and relative water contents in both unfertilized and fertilized seedlings under ambient CO_2_, and in unfertilized seedlings under elevated CO_2_ (Table 1, Fig. 2; root water content, g). However, under elevated CO_2_ the absolute water content was higher in defoliated than intact seedlings at high nutrients (Fig. 2).

Main root length in all seedlings varied between 11 and 57 cm, and increased by elevated CO_2_ (Table 1); under the treatment the root length was approximately 38.7 ± 0.9 cm, whereas under ambient CO_2_, it was 35.1 ± 0.9 cm. The longest roots, on average 38.9 ± 0.8 cm, were recorded in unfertilized seedlings. In fertilized seedlings, the mean root length was 35.0 ± 0.8 cm. Defoliation had no effect on root length.

### Absolute and relative root carbohydrates (g DW and % DW)

**Figure 3:**
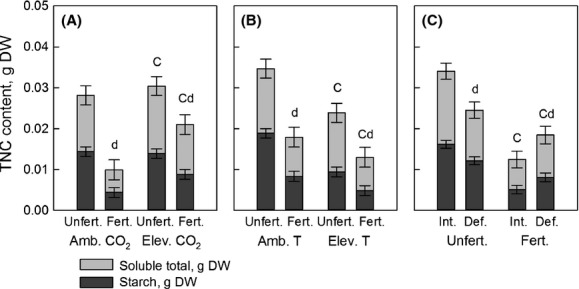
Contents (g dry weight, DW) of total nonstructural carbohydrates (TNCs) in fine roots of silver birch seedlings. These are divided into insoluble starch and soluble carbohydrates, sucrose, fructose, and glucose. Estimated marginal means with ±SE are presented as histograms in different treatment combinations (A: CO_2_ × fertilization; B: temperature × fertilization; and C: fertilization × defoliation). In A and B, the letter “C” indicates a statistically significant (*P* < 0.05, based on the Tukey's test) difference between ambient and elevated climate treatment, and the letter “d”, unfertilized and fertilized seedlings. In 3C, the letter “C” indicates a statistically significant difference between unfertilized and fertilized seedlings, and the letter “d”, defoliated and intact seedlings. Fine root samples for the analysis were collected in late March 2003.

In all fine root samples, total nonstructural carbohydrates (TNC; the combination of starch, sucrose, fructose, and glucose) were in the range 0.003–0.061 g DW or 0.5–7.1% DW. In comparison to ambient CO_2_, elevated CO_2_ increased the absolute root TNCs by 25% (ambient CO_2_; 0.019 ± 0.002 g DW vs. elevated CO_2_; 0.026 ± 0.002 g DW; Table 2, Fig. 3A). Instead, elevated temperature lowered the TNC contents (Fig. 3B). Fertilization markedly decreased both the absolute and relative TNCs, affecting stronger the absolute contents under ambient than elevated temperature or CO_2_ (Table 2, Figs 3A and B). Irrespective of climate treatments, the TNCs in unfertilized seedlings were higher than in fertilized seedlings (Fig. 3A and B). Defoliation increased both the absolute and relative TNCs in fertilized seedlings, whereas the response was the opposite, but weaker in unfertilized counterparts (Fig. 3C).

**Table 2:** Summary of linear mixed model analysis results for the effects of temperature, CO_2_, fertilization, and defoliation and their interactions on fine root carbohydrates of silver birch seedlings

Source	Nonstructural carbohydrates of roots															

	Total: combination of starch, glucose, fructose, and sucrose								Starch							
	
	0.02 ± 0.001 g DW (0.003–0.061 g DW)				3.4 ± 0.24% DW (0.5–7.1% DW)				0.011 ± 0.001 g DW (<0.001–0.03 g DW)				1.56 ± 0.14% DW (0–4.1% DW)			
			
	N df	D df	*F*	*P*	N df	D df	*F*	*P*	N df	D df	*F*	*P*	N df	D df	*F*	*P*
CO_2_	1	11.9	5.20	**0.042**	1	11.7	0.34	0.573	1	11.9	1.82	0.203	1	11.8	1.25	0.286
Temperature (T)	1	11.9	7.27	**0.020**	1	11.7	1.55	0.238	1	11.9	19.18	**<0.001**	1	11.8	0.02	0.904
CO_2_ × T	1	11.9	1.10	0.314	1	11.7	0.96	0.346	1	11.9	0.28	0.604	1	11.8	1.64	0.225
Fertilization (F)	1	32.5	71.66	**<0.001**	1	32.1	53.83	**<0.001**	1	32.5	91.05	**<0.001**	1	32.4	30.25	**<0.001**
Defoliation (D)	1	32.5	1.18	0.286	1	32.1	0.22	0.642	1	32.5	0.42	0.521	1	32.4	<0.01	0.952
CO_2_ × F	1	32.5	7.28	**0.011**	1	32.1	0.91	0.347	1	32.5	9.58	**0.004**	1	32.4	0.14	0.712
CO_2_ × D	1	32.5	0.85	0.363	1	32.1	0.36	0.550	1	32.5	0.46	0.505	1	32.4	0.38	0.540
T × F	1	32.5	3.28	**0.080**	1	32.1	3.12	**0.087**	1	32.5	14.30	**<0.001**	1	32.4	<0.01	0.967
T × D	1	32.5	0.11	0.744	1	32.1	0.18	0.676	1	32.5	0.01	0.924	1	32.4	0.16	0.688
F × D	1	32.5	22.47	**<0.001**	1	32.1	3.70	**0.063**	1	32.5	19.58	**<0.001**	1	32.4	2.57	0.119
CO_2_ × T × F	1	32.5	0.89	0.352	1	32.1	1.02	0.320	1	32.5	2.00	0.167	1	32.4	0.43	0.515
CO_2_ × T × D	1	32.5	2.66	0.112	1	32.1	0.28	0.600	1	32.5	2.15	0.152	1	32.4	0.37	0.545
CO_2_ × F × D	1	32.5	0.29	0.594	1	32.1	0.04	0.839	1	32.5	2.40	0.131	1	32.4	0.23	0.636
T × F × D	1	32.5	0.24	0.626	1	32.1	<0.01	0.996	1	32.5	0.16	0.696	1	32.4	0.11	0.743
CO_2_ × T × F × D	1	32.5	0.09	0.770	1	32.1	0.06	0.810	1	32.5	0.43	0.515	1	32.4	0.03	0.869

	Soluble tot.: glucose, fructose, sucrose				Glucose				Fructose				Sucrose			
				
	1.8 ± 0.12% DW (0.5–4.6% DW)				1.04 ± 0.05% DW (0.3–1.8% DW)				0.64 ± 0.07% DW (0–3.5% DW)				0.13 ± 0.05% DW (0–2.4% DW)			
CO_2_	1	11.7	0.01	0.938	1	11.7	0.11	0.751	1	12.1	0.82	0.383	1	12.3	1.87	0.196
Temperature (T)	1	11.7	4.45	**0.057**	1	11.7	1.51	0.243	1	12.1	0.17	0.689	1	12.3	3.76	**0.076**
CO_2_ × T	1	11.7	0.41	0.533	1	11.7	0.05	0.820	1	12.1	1.15	0.304	1	12.3	2.89	0.114
Fertilization (F)	1	32.1	53.22	**<0.001**	1	32.1	14.96	**<0.001**	1	33	32.80	**<0.001**	1	33.9	0.07	0.792
Defoliation (D)	1	32.1	0.85	0.364	1	32.1	0.21	0.647	1	33	0.64	0.430	1	33.9	0.35	0.558
CO_2_ × F	1	32.1	1.75	0.196	1	32.1	0.63	0.433	1	33	0.31	0.582	1	33.9	0.6	0.445
CO_2_ × D	1	32.1	0.14	0.714	1	32.1	1.06	0.310	1	33	0.19	0.666	1	33.9	0.78	0.385
T × F	1	32.1	9.83	**0.004**	1	32.1	0.06	0.814	1	33	<0.01	0.977	1	33.9	0.11	0.743
T × D	1	32.1	0.09	0.772	1	32.1	0.31	0.584	1	33	0.49	0.489	1	33.9	0.3	0.588
F × D	1	32.1	3.09	**0.088**	1	32.1	1.37	0.250	1	33	1.23	0.275	1	33.9	0.66	0.424
CO_2_ × T × F	1	32.1	1.33	0.257	1	32.1	0.27	0.609	1	33	1.01	0.323	1	33.9	0.15	0.698
CO_2_ × T × D	1	32.1	0.10	0.752	1	32.1	0.94	0.339	1	33	0.05	0.832	1	33.9	0.5	0.484
CO_2_ × F × D	1	32.1	0.89	0.353	1	32.1	0.07	0.798	1	33	0.04	0.848	1	33.9	0.6	0.444
T × F × D	1	32.1	0.10	0.757	1	32.1	1.40	0.245	1	33	0.24	0.626	1	33.9	0.75	0.393
CO_2_ × T × F × D	1	32.1	0.36	0.553	1	32.1	2.14	0.153	1	33	0.11	0.747	1	33.9	0.86	0.361

All the concentrations are presented as percentage of dry mass (% DW). The total nonstructural carbohydrates (including starch, sucrose, fructose, and glucose) and starch singly are presented as g of dry mass (g DW). The mean and ±SE values and the range of g DW or % DW in all samples are given. The significant *P* values (<0.1) are given in bold. N df, numerator degrees of freedom; D df, denominator degrees of freedom.

Across all root samples, the combined relative concentration of soluble carbohydrates (sucrose, fructose, and glucose) ranged between 0.5% and 4.6% DW, and decreased by fertilization (Table 1; concentration in fertilized seedlings 1.3 ± 0.2% DW vs. unfertilized seedlings 2.2 ± 0.2% DW). When analyzed separately, concentrations of the two common monosaccharides, glucose and fructose (mean concentrations 1.0 ± 0.1% DW and 0.6 ± 0.1% DW, respectively), were significantly lower in fertilized than unfertilized seedlings, whereas sucrose responded to temperature elevation (Table 2). Sucrose concentration was eight times higher under elevated than ambient temperature (0.24 ± 0.08% DW vs. 0.03 ± 0.08% DW, respectively).

Defoliation alone had no significant effect on the specific root carbohydrates. However, the absolute content of insoluble storage carbohydrate starch was 59% higher in defoliated than intact seedlings at high nutrients (0.008 ± 0.001 g DW vs. 0.005 ± 0.001 g DW, respectively). At low nutrients, defoliation decreased the starch content by 25% (intact 0.016 ± <0.001 g DW vs. defoliated 0.012 ± <0.001 g DW) (Table 2, Fig. 3C). Fertilizing decreased both the absolute and relative amounts of starch; however, the absolute starch content responded to fertilizer application stronger under ambient than elevated temperature; under ambient temperature the content at high nutrients was 0.008 ± 0.0012 g DW, whereas at low nutrients it was 0.019 ± 0.0011 g DW. Similar responses to fertilization were found under elevated CO_2_ (Table 2); although elevated CO_2_ slightly increased the absolute starch content, fertilization decreased it (Fig. 3A).

## Discussion

Previous research has reported that increases in soil nutrient availability alter above- and belowground carbon gain and growth in many tree species (see, e.g., Haynes and Gower 1995; Kleczewski et al. 2012). Our results confirm that fertilization reduced belowground resource allocation in silver birch seedlings by decreasing the main root length, the proportion of fine roots, and both the absolute and relative concentrations of nonstructural carbohydrates, TNCs. This goes along with the optimal partitioning theory predicting that plants optimize biomass allocation into organs providing growth-limiting resources (Bloom et al. 1985; Kobe et al. 2010). Thus, root proliferation and increase in their carbohydrate concentration under nutrient poor soil reveal an allocation pattern to support formation of nutrient absorptive surface (e.g., Kobe et al. 2010). Converse responses under nutrient-rich soil imply resource allocation toward shoots due to light and assimilated carbon becoming growth-limiting factors (Tilman 1990). Furthermore, the reasons for lower carbohydrate (especially starch) accumulation in roots (Fig. 3) possibly are delays in both stem growth termination and winter hardening at ample nutrients (see Laitinen et al. 2004). Also the pioneering fast growth habit of the tree species may explain our results; birches typically allocate most of the photosynthates to stem and leaf expansion in their early seedling years, which leads to lower amounts of carbohydrates directed to root growth and storage (Gaugher et al. 2005). In fertile soil where the needs for micro- and macronutrients are met, this growth habit will magnify aboveground productivity, as shown in Huttunen et al. (2007).

Interestingly and against our original hypothesis, not only high nutrients but also low nutrients with defoliation caused a decrease in the proportion of fine roots of birch seedlings (Fig. 2). At the same time, the absolute contents of TNCs (combination of starch, sucrose, glucose, and fructose) and insoluble starch (Fig. 3) increased in roots of defoliated and fertilized seedlings. Foliage damage typically shifts resource partitioning to aboveground production (Anttonen et al. 2002; Barry et al. 2012), which leads to decreased belowground biomass and carbohydrate accumulation (e.g., Markkola et al. 2004; Frost and Hunter 2008; Eyles et al. 2009). This kind of pattern has been recorded in trees irrespective of nutrient availability (Eyles et al. 2009). Our contrasting findings on absolute TNCs in fertilized seedlings suggest that defoliated plants were able to sustain intensified photosynthesis later to the season than plants without defoliation (see also Perry 1971; Wargo 1991; Nilsen and Wielgolaski 2001). As a result of naturally terminated shoot height growth by declining day length (the demand for assimilates in shoots weakens) and stimulated photosynthesis in leaves following defoliation (see Huttunen et al. 2007), the carbon flux has been directed into the root stores before leaf senescence and abscission in autumn. This explains the differences in both the proportions and carbohydrate concentrations of fine roots in differently fertilized and defoliated seedlings compared with their counterparts. In defoliated seedlings, the ability to sustain photosynthesis in late season not only indicates alteration in root growth and carbohydrate gain but also delay in the winter hardening process (Perry 1971; Wargo 1978).

Elevation in CO_2_ increased both the absolute fine root TNCs and the total root mass, especially in fertilized seedlings (Figs. 2 and 3). The mechanism behind these increases may be stimulated photosynthesis (Huttunen et al. 2007); the increased amounts of low molecular weight carbohydrates (such as sucrose, glucose, and fructose) were allocated to provide substrates for both insoluble cell wall components (cellulose, hemicellulose, pectin, and lignin; Gibson 2012) and storing organs; this increases root mass and length, as well as absolute contents of TNCs, including starch. Here, the storage carbohydrate pools are important in determining trees' sensitivity to disturbances, such as defoliation with respect to their easy mobilization (Palacio et al. 2011).

Elevated temperature significantly decreased both the proportion of fine roots in defoliated seedlings and the absolute contents of root TNCs and starch (Fig. 3B). However, the carbohydrates in fertilized seedlings responded to temperature elevation substantially milder. General impacts of warming include decreases in root masses (Kuokkanen et al. 2004), as well as their TNC concentrations with great variability between different tree species (Shi et al. 2006; Li et al. 2008). These reductions indicate that carbon demand exceeds supply in warmer environments due to increased root respiration (Atkin et al. 2000) and enhanced aboveground growth (Kuokkanen et al. 2004; Huttunen et al. 2007); also intensified foliar transpiration increases water flow in fine roots and thus dilutes root carbohydrates (Fiscus 1975; Wayne et al. 1998). At the same time, relative concentration of sucrose in fine roots showed increasing response to temperature elevation. Many photosynthates are known to function as secondary signalers with the ability to promote or inhibit plant growth and development in response to biotic and abiotic stresses (Hammond and White 2008; Wind et al. 2010; Mäenpää et al. 2013). Related to growth regulation, Suriyagoda et al. (2012) reported that species with fast relative growth rates consume more phosphorus under elevated temperature, and the uptake of the element is determined by root length rather than unit root surface area. Furthermore, Hammond and White (2008) found that phosphorus starvation in plants leads to relocation of sucrose to the roots increasing their growth and thus optimizing phosphorus uptake. This possibly explains the increased sucrose concentration in those birch seedlings that were subjected to elevated temperature.

As starch, sucrose, glucose, and fructose are involved in cell wall synthesis (Gibson 2012), and their combined contents were low in fine roots of seedlings at high temperatures, it is logical that the proportion of fine roots also decreased under elevated temperature (Table 1). For instance, Kuokkanen et al. (2004) reported low biomasses in roots of silver birch seedlings that were subjected to high temperature. The consequent shallow root systems will in theory diminish the ability of seedlings to tolerate dry periods, and thus increase the risk of plant mortality, as the role of fine roots is to absorb water and nutrients from the soil (e.g., Lukac and Godbold 2011). However, root water content in birch seedlings was not affected by temperature; this possibly derives from daily irrigation and thus improved water supply during the growing season 2002. Instead, the root water content increased in seedlings at high nutrients and CO_2_. Typically, nutrient status of plants affects cell water relations by changing stomatal sensitivity, increasing water use efficiency, and altering turgor and osmotic adjustments (Augé et al. 1990). Moreover, if the seedlings were defoliated and grown at low nutrients, the root water content decreased under both ambient and elevated CO_2_ (Fig. 2). Quentin et al. (2011) have demonstrated that foliage damage magnifies aboveground transpiration and soil-to-leaf hydraulic conductance reducing root water content. Thus, increased water flow through transpiration and overcompensated aboveground growth (see Huttunen et al. 2007) explains the outcome in defoliated birch seedlings.

As a summary, fertilization alone negatively affected root systems and their carbohydrate concentrations in all silver birch seedlings, although defoliation slightly increased the absolute TNCs and starch at high nutrients. This possibly resulted from stimulated photosynthesis, which was induced by defoliation (see Huttunen et al. 2007), and which continued later to the season due to complex source–sink and signaling adjustments. Moreover, elevation in temperature decreased the proportion of fine roots in defoliated seedlings and the root TNCs in unfertilized seedlings. This is possibly related to increased root respiration and enhanced aboveground growth through temperature elevation (Huttunen et al. 2007). However, fertilization increased both root TNCs and mass under elevated CO_2_, which possibly is linked with intensified photosynthesis (Huttunen et al. 2007). It seems that increases in temperature, CO_2_, and nutrient availability have no interactive effects on morphology or carbohydrate gain in roots of defoliated silver birch seedlings. Nevertheless, it is difficult to estimate the overall impacts of climate change on belowground carbon dynamics and growth in deciduous trees due to the complexity and partly mitigating nature of the effects recorded. As much as ample nutrients increase aboveground productivity and thereby decrease root storage and growth, defoliation or elevations in temperature or CO_2_ tend to alleviate these responses in birch seedlings.
